# Antioxidant Efficacy of Hwangryunhaedok-tang through Nrf2 and AMPK Signaling Pathway against Neurological Disorders In Vivo and In Vitro

**DOI:** 10.3390/ijms25042313

**Published:** 2024-02-15

**Authors:** Su-Jin Bae, Won-Yung Lee, Seon Been Bak, Seung Jin Lee, Su-Jin Hwang, Geun-Woo Kim, Byung-Soo Koo, Sun-Dong Park, Hye-Hyun Yoo, Choon-Ok Kim, Hyung Won Kang, Tae-Woo Oh, Young Woo Kim

**Affiliations:** 1 School of Korean Medicine, Dongguk University, Gyeongju 38066, Republic of Korea; realsujin@naver.com (S.-J.B.); wonyung21@naver.com (W.-Y.L.); skyblue014@gmail.com (S.B.B.); lovewing85@naver.com (S.J.L.); kgwoo86@hanmail.net (G.-W.K.); koobs1009@gmail.com (B.-S.K.); sundong@dongguk.ac.kr (S.-D.P.); 2 College of Korean Medicine, Wonkwang University, Iksan 54538, Republic of Korea; onp21@daum.net; 3 Korean Medicine (KM)-Application Center, Korea Institute of Oriental Medicine (KIOM), Daegu 41062, Republic of Korea; 4 College of Pharmacy, Hanyang University, Ansan 1558, Republic of Korea; yoohh@hanyang.ac.kr; 5 Department of Clinical Pharmacology and Clinical Trials Center, Severance Hospital, Yonsei University Health System, Seoul 03722, Republic of Korea; delivery98@yuhs.ac; 6 Department of Korean Convergence Medical Science, University of Science & Technology (UST), Daejeon 34054, Republic of Korea

**Keywords:** Hwangryunhaedok-tang, baicalein, Alzheimer’s disease, Nrf2, AMPK

## Abstract

Alzheimer’s disease (AD) is a representative cause of dementia and is caused by neuronal loss, leading to the accumulation of aberrant neuritic plaques and the formation of neurofibrillary tangles. Oxidative stress is involved in the impaired clearance of amyloid beta (Aβ), and Aβ-induced oxidative stress causes AD by inducing the formation of neurofibrillary tangles. Hwangryunhaedok-tang (HHT, Kracie K-09^®^), a traditional herbal medicine prescription, has shown therapeutic effects on various diseases. However, the studies of HHT as a potential treatment for AD are insufficient. Therefore, our study identified the neurological effects and mechanisms of HHT and its key bioactive compounds against Alzheimer’s disease in vivo and in vitro. In a 5xFAD mouse model, our study confirmed that HHT attenuated cognitive impairments in the Morris water maze (MWM) test and passive avoidance (PA) test. In addition, the prevention of neuron impairment, reduction in the protein levels of Aβ, and inhibition of cell apoptosis were confirmed with brain tissue staining. In HT-22 cells, HHT attenuates tBHP-induced cytotoxicity, ROS generation, and mitochondrial dysfunction. It was verified that HHT exerts a neuroprotective effect by activating signaling pathways interacting with Nrf2, such as MAPK/ERK, PI3K/Akt, and LKB1/AMPK. Among the components, baicalein, a bioavailable compound of HHT, exhibited neuroprotective properties and activated the Akt, AMPK, and Nrf2/HO-1 pathways. Our findings indicate a mechanism for HHT and its major bioavailable compounds to treat and prevent AD and suggest its potential.

## 1. Introduction

Globally, the number of dementia patients is predicted to increase from 57.4 million in 2019 to 152.8 million in 2050, considering the aging population [1]. Alzheimer’s disease, the major cause of dementia, shows a specific neuropathological characteristic: extracellular depositions of diffuse and neuritic plaques, formed by higher-order fibrils of amyloid beta (Aβ) released from cleaved β-amyloid precursor protein (APP) and the intracellular accumulation of neurofibrillary tangles composed of aberrantly hyperphosphorylated tau protein [2]. Amyloid beta aggregation is responsible for a decreased synaptic activity and increased synapse loss, impaired cerebral capillary blood flow, and pathological spread of tau to other cortical regions by the hyperphosphorylation of tau and stimulation of other pathways. Therefore, Aβ accumulation is the main initiator of the pathological process of AD, which can lead to neurodegeneration by neuroinflammation and amyloid-facilitated tauopathy [3]. Tau aggregates are mainly located in the medial temporal lobe after the age of 60 years; they begin in the entorhinal cortex, spread to the hippocampus, and then disperse beyond the limbic area as dementia progresses [4].

The pathogenesis of AD is influenced by a multitude of factors, encompassing not only Aβ accumulation and Tau spread, but also neuroinflammation, alterations in the vessels, aging, and the dysfunction of the glymphatic system [5]. Among these factors, the association between oxidative stress and the etiology of AD is noteworthy [6,7,8]. Oxidative stress is involved in Aβ clearance, and Aβ-induced oxidative stress causes neurofibrillary tangles to form by modification of the tau conformation, which is a critical factor in the pathogenesis of AD [9]. Dysfunction caused by oxidative stress in the mitochondria, which influences the cell’s bioenergetic state and redox environment, affects brain amyloidosis and the pathogenesis of AD [10]. Ultimately, the Aβ and tau interaction promote neurodegeneration and cognitive decline [11]. Therefore, the use of agents to inhibit oxidative stress might be a strategy for blocking the pathological process of neurological disorders.

Hwangryunhaedok-tang (HHT) is a traditional herbal medicine prescription in Korea and is called the Huanglian jiedu decoction in China and Orengedoku-to in Japan. Hwangryunhaedok-tang is a combination of four medicinal herbs (Coptidis Rhizoma, Phellodendron bark, Scutellariae Radix, and Gardeniae Fructus) in a 1:1:1:1 ratio [12]. HHT has been reported to improve chronic obstructive pulmonary disease [13], atherosclerosis [14], and arthritis [15]. In addition, HHT attenuated oxidative stress and decreased hippocampal amyloid beta (Aβ) in an Aβ_25–35_-induced Alzheimer’s disease model [16].

HHT identified 14 marker compounds through an analysis using ultra-high-performance liquid chromatography coupled with quadrupole-Orbitrap high-resolution mass spectrometry [17]. Among them, baicalein is the major bioactive compound of Scutellariae Radix (the dried root of Scutellaria baicalensis Georgi) among the medicinal herbs that comprise HHT [18] and has anticancer, anti-inflammatory, and antioxidant effects [19]. Baicalein has potential neuroprotective effects against ischemia or stroke-induced neuronal cell death [20] and is a potent neuroprotective agent against AD and PD associated with the gradual damage of neuronal cells and nervous system dysfunction [21]. Although the effects of HHT and baicalein on AD have been reported in some studies, their antioxidant efficacy and underlying mechanisms have not been fully elucidated. Therefore, we verified the effects of HHT (K-09^®^), which was used as a standardized pharmaceutical of HHT manufactured by Kracie Pharma, Ltd. (Minato-ku, Tokyo, Japan), on neurological disorders in vivo and in vitro.

## 2. Results

### 2.1. HHT Attenuated the Cognitive Impairments in Six-Month-Old 5xFAD Mice

**Figure 1:**
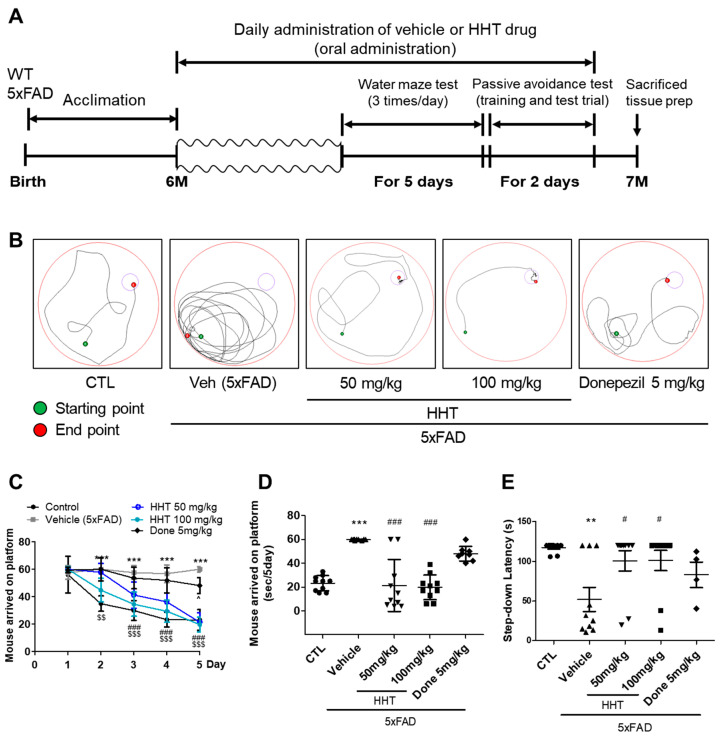
Effects of HHT, resulting in reduced cognitive impairments in 5xFAD transgenic AD mice. (**A**) Schematic timeline of experiment; (**B**) representative tracks of the test during the probe trial on day 5; (**C**) escape latency for 5 days; *** *p* < 0.001 (CTL vs. Veh), ^###^ *p* < 0.001 (Veh vs. HHT 100 mg/kg), and ^$$^ *p* < 0.01 and ^$$$^ *p* < 0.001 (Veh vs. HHT 100 mg/kg). (**D**) Mice arriving at the platform in a Morris water maze (MWM) test and (**E**) step-through latency in passive avoidance (PA) test. Experimental data represent the means ± S.E.M. ** *p* < 0.01 and *** *p* < 0.001 (vs. CTL); ^#^ *p* < 0.05 and ^###^ *p* < 0.001 (vs. Veh). Veh, 5xFAD mice; Done, donepezil.

HHT was evaluated regarding its protective effects against cognitive impairment in AD mouse models, which were transgenic mice expressing a 5xFAD mutation (5xFAD mice) [22]. A vehicle solution and HHT (50 or 100 mg/kg/day) were administered orally (p.o.) to six-month-old WT and 5xFAD mice for four weeks, followed by a behavioral test (Figure 1A). We performed the MWM test to investigate the effects of HHT on the cognitive function of 5xFAD mice. The track of the mice is shown in Figure 1B; normal mice showed relatively fast and good learning, whereas 5xFAD mice showed poor learning and memory. During the five days of training and testing, the mice administered HHT showed a marked decrease in the average escape latency and distance traveled compared with those of the vehicle group, with statistical significance observed on the fifth day (Figure 1C,D). These differences in memory between the groups were also evident in the swimming trajectories on the sixth day (Figure 1B). Next, a PA test was performed to assess how long a mouse could remember its location (Figure 1E). As shown in Figure 1E, HHT mice had a significantly increased step-through latency compared to the 5xFAD mice. In the results of the MMW and PA experiments, HHT showed a good effect on anti-AD drugs, and donepezil, used as a positive control, also showed a generally good effect. Overall, these results indicate that HHT rescued cognitive function impairments in 5xFAD mice.

### 2.2. Protective Effects of HHT on Neuronal Damage in 5xFAD Mice

**Figure 2:**
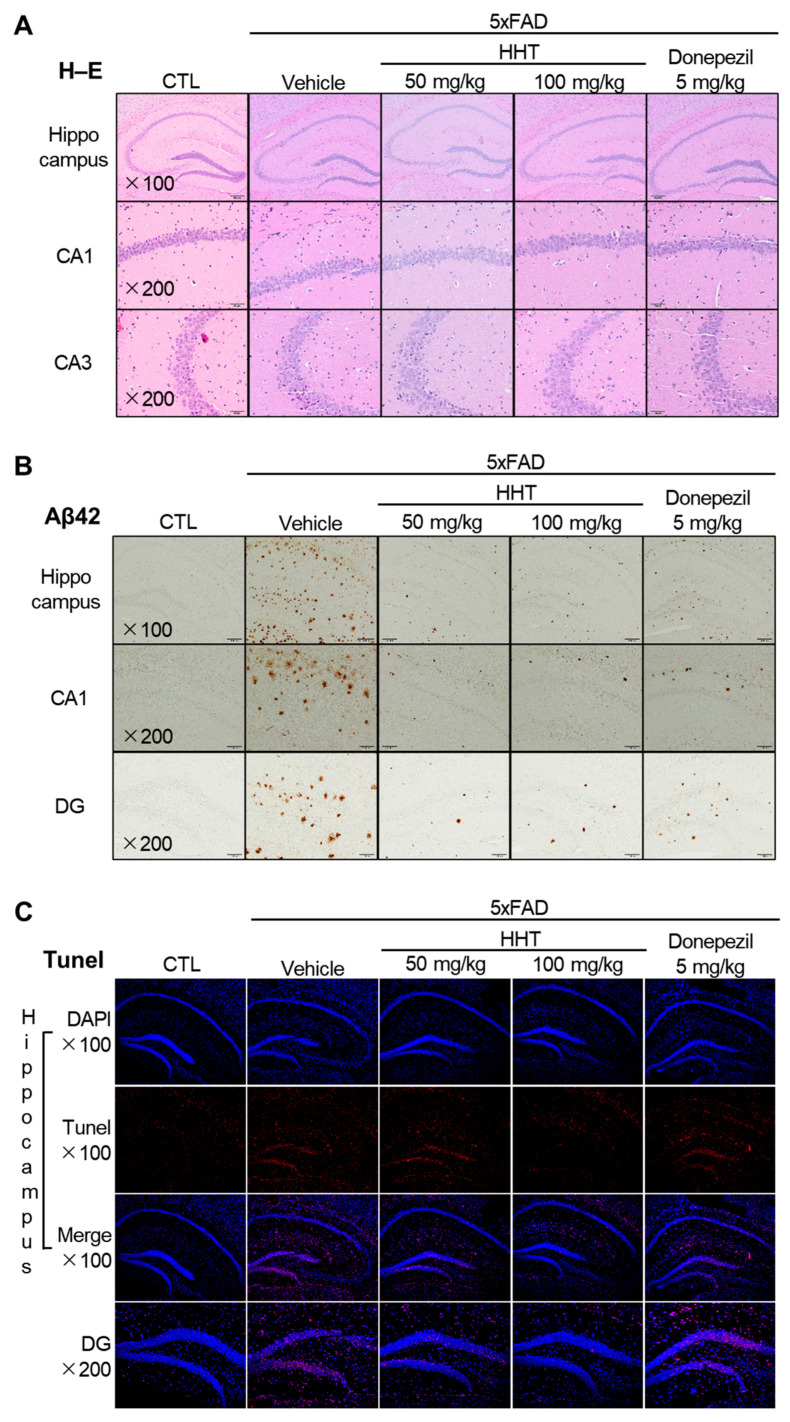
HHT decreased neuronal damage, Aβ accumulation, and the expression in hippocampus of 5xFAD mice. (**A**) Representative images of H–E staining in the CA1 and CA3 regions of the hippocampus among experimental groups. Images were taken through a compound light microscope using a 100× (upper) and 200× (middle and lower) objective. (**B**) Representative images of Aβ protein levels in the CA1 and DG hippocampal regions among experimental groups. Images were taken through a compound light microscope using a 100× (upper) and 200× (middle and lower) objective. (**C**) Representative immunofluorescence images for detection of apoptosis cell by TUNEL staining assay (red) and Nuclei were stained blue (DAPI) in the hippocampus. Images were taken through a compound light microscope using a 100× and 200× (DG region) objective.

To evaluate the effects of HHT on neuronal damage in the mouse brain, histopathological changes were observed through H–E staining. A normal form of morphology in the hippocampus and cortex was determined as the basis for judgment. As a result, in the Alzheimer’s dementia modeling mice, 5xFAD mouse brain tissue from the pyramidal neurons of the cornu ammonis (CA1) and dentate gyrus (DG) of the hippocampus showed deformed forms, such as nuclear fragmentation, clumping, and pyknosis due to damage, and the number of cells decreased. The density of the surrounding tissues decreased. Conversely, it was significantly recovered by the administration of HHT or donepezil (Figure 2A).

We analyzed the expression of Aβ plaques in the brain sections of 5xFAD mice. The number of Aβ plaques was quantified in the hippocampus and CA1 and DG areas of the hippocampus in six-month-old 5xFAD mice with the HHT or vehicle. The number of Aβ plaques was significantly higher in the hippocampus, CA1, and DG of vehicle-treated 5xFAD mice, whereas treatment with HHT or donepezil reduced this increase in the plaque numbers of 5xFAD mice (Figure 2B). 

To confirm that apoptotic cell death was reduced by HHT, TUNEL staining was performed. Apoptotic cell death was visualized by TUNEL staining (red) and counterstaining with DAPI (blue) (Figure 2C). The number of TUNEL-positive cells, which rose sharply in 5xFAD mice, was significantly reduced in the hippocampus and DG after HHT administration (Figure 2C).

### 2.3. HHT Attenuates tBHP-Induced Cytotoxicity, ROS Generation, and Mitochondrial Dysfunction in HT-22 Cells

**Figure 3:**
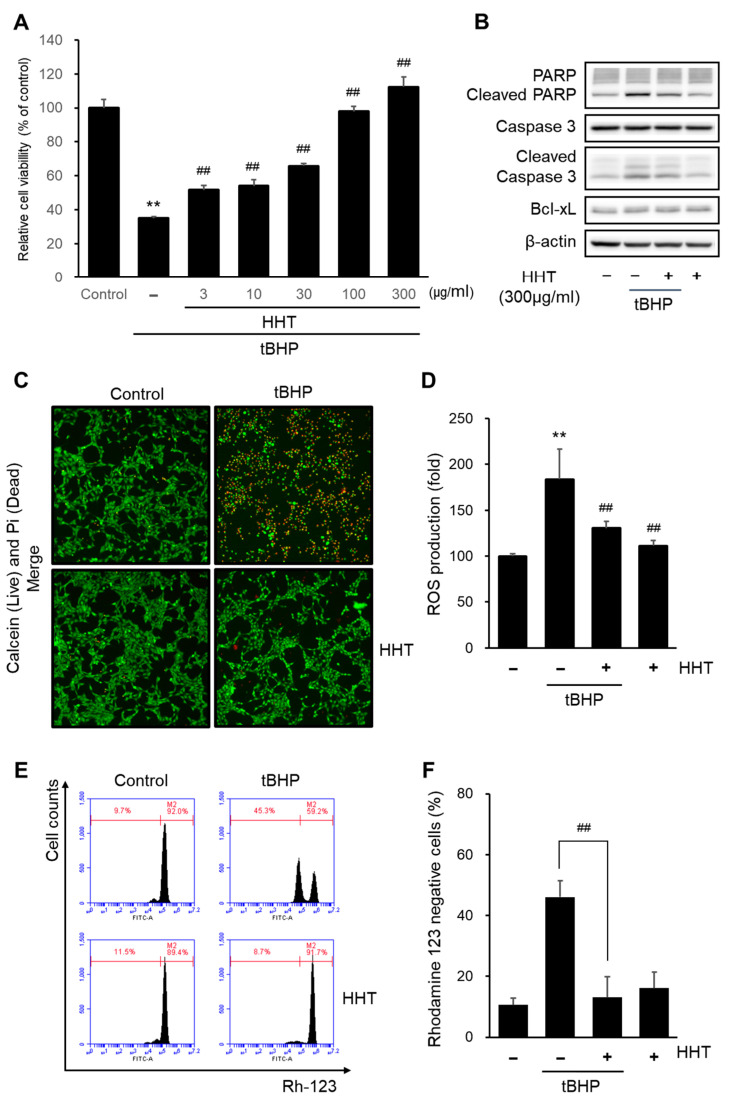
Neuroprotective effect of HHT on tBHP-induced cytotoxicity, ROS generation, and mitochondrial dysfunction. (**A**) Cell viability was examined using the MTT assay. HT-22 cells were treated with HHT (3, 10, 30, 100, and 300 μg/mL) for 1 h, followed by incubation with 100 μm tBHP for 6 h. Data are presented as the means ± SDs of three replicate experiments (** *p* < 0.01, significant difference compared to control; ^##^ *p* < 0.01, significant difference compared to tBHP). (**B**) HT-22 cells were incubated with or without 300 μg/mL HHT for 1 h, then incubated with 100 μm tBHP for 6 h. Immunoblot analysis for apoptosis-associated proteins was performed with HT-22 cell lysates. (**C**) Cell viability was measured using a fluorescence microscope after staining with calcein AM (0.5 μg/mL) and propidium iodide (0.5 μg/mL) for 30 min. (**D**) HT-22 cells were treated as described in Figure 3B. (**D**) Cellular ROS generation was measured using a DCFH-DA assay kit. (**E**,**F**) The fluorescence intensity of rhodamine 123 in the mitochondrial inner membrane was analyzed using a flow cytometer. Rhodamine 123 (0.05 μg/mL) staining was carried out for 1 h. Data are presented as the means ± SDs of three replicate experiments (** *p* < 0.01, significant difference compared to control; ^##^ *p* < 0.01, significant difference compared to tBHP).

To study the neuroprotective effects of HHT in vitro, we performed an MTT assay. Tert-butylhydroperoxide (tBHP)-induced oxidative stress in HT-22 cells was treated with HHT (3, 10, 30, 100, and 300 µg/mL). The stimulation of tBHP significantly reduced cell viability compared to that of the control group. In contrast, treatment with HHT in tBHP markedly improved the cell viability in a dose-dependent manner (Figure 3A). The cell viability was most improved at 300 ug/mL of HHT, which was used in the experiments. To further investigate the neuroprotective effects of HHT, we confirmed the expressions of apoptosis-related proteins such as PARP, caspase-3, and Bcl-xL (Figure 3B). Treatment with tBHP increased the cleaved PARP and cleaved caspase-3 levels, but treatment with HHT suppressed the expression of these proteins. Additionally, a decrease in apoptotic cells induced by HHT was observed in calcein AM and PI staining (Figure 3C).

We also determined the antioxidant effect of HHT by measuring intracellular ROS levels. tBHP significantly elevated the intracellular ROS generation, whereas 300 μg/mL of HHT did not. A pretreatment with 300 μg/mL HHT reduced the tBHP-induced intracellular ROS generation (Figure 3D). Mitochondrial dysfunction was verified by rhodamine 123, which is distributed in the mitochondrial matrix, according to the MMP. tBHP significantly increased rhodamine 123 negative cells, whereas 300 μg/mL HHT did not. A pretreatment with 300 μg/mL HHT decreased the rhodamine 123 negative cells caused by tBHP (Figure 3E,F). These results indicate that HHT exerts a neuroprotective effect by reducing the ROS production and mitochondrial dysfunction.

### 2.4. HHT Alleviates Cytotoxicity by Activating the Nrf2/HO-1 Signaling Pathway

**Figure 4:**
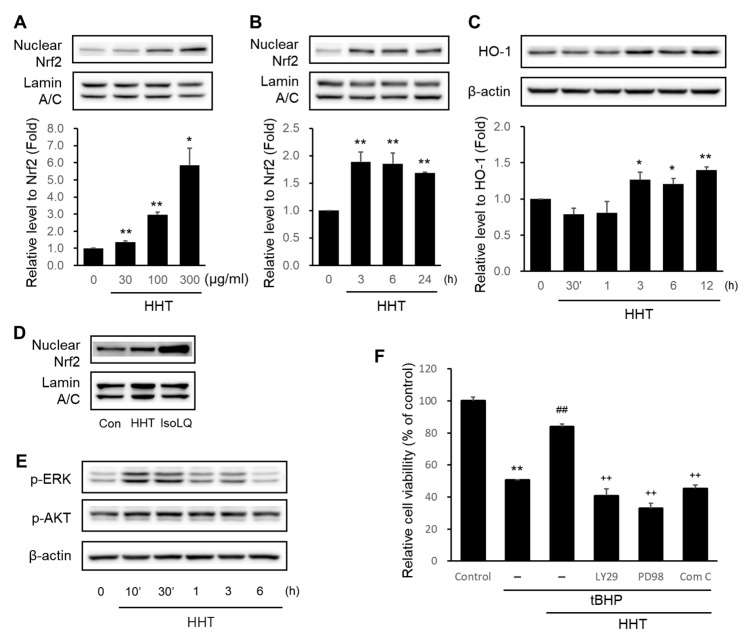
Effects of HHT on the activation of the Nrf2/HO-1 signaling pathway. The HT-22 cells were treated with (**A**) 30, 100, and 300 μg/mL HHT for 6 h and (**B**) 300 μg/mL HHT for the indicated time. Subsequently, nuclear fractionation was performed for the immunoblotting analysis of the accumulation of Nrf2 in the cell nucleus. Lamin A/C was used for the immunoblotting analysis for equal loading control of cell nucleus proteins. (**C**) Immunoblotting analysis of HO-1 was performed with lysates of HT-22 cells treated with 300 μg/mL HHT for 30 min to 12 h. Expression of Nrf2 and HO-1 are presented as means ± SD of three replicate experiments (* *p* < 0.05; ** *p* < 0.01, significant difference compared to control). (**D**) Immunoblotting analysis of Nrf2 was performed using lysates of HT-22 cells treated with 300 μg/mL HHT and 20 μM IsoLQ for 3 h each. (**E**) Immunoblotting analyses of p-ERK and p-Akt were performed with HT-22 cells lysates treated with 300 μg/mL HHT for 10 min to 6 h. (**F**) HT-22 cells were treated or not with LY294002 (30 μM), PD98059 (30 μM), and compound C (5 μM) for 1 h, followed by incubation with 300 μg/mL HHT for 1 h. Additionally, after incubation with 100 μm tBHP for 6 h, the MTT assay was performed. Data are presented as means ± SDs of three replicate experiments (** *p* < 0.01, significant difference compared to control; ^##^ *p* < 0.01, significant difference compared to tBHP; ^++^ *p* < 0.01, significant difference compared to tBHP + HHT).

To elucidate the mechanisms underlying the neuroprotective effects of HHT, we verified representative pathways involved in antioxidant properties, particularly the Nrf2/HO-1 signaling pathway. The highest Nrf2 expression in the nucleus of HT-22 cells was caused by 300 μg/mL HHT (Figure 4A). Treatment with 300 μg/mL HHT maximally elevated the Nrf2 expression in the cell nucleus at 3 h and continued to enhance the Nrf2 expression up to 24 h (Figure 4B). HO-1, a detoxification enzyme expressed in the ARE by Nrf2, was significantly elevated at 3 h in HT-22 cells treated with 300 μg/mL HHT and was most expressed at 12 h (Figure 4C). The standard antioxidant compound, isoliquiritigenin (IsoLQ), was used as a positive control to confirm the expression of Nrf2 with HHT. Previous studies confirmed that 20 μM isoLQ leads to a maximal expression of Nrf2 [23] (Figure 4D).

Accordingly, it was demonstrated that HHT might exert a neuroprotective effect by affecting the signaling pathways that interact with Nrf2.

Furthermore, we confirmed signaling pathways such as MAPK/ERK, PI3K/Akt [24], and AMPK [25,26,27] that are collaboratively linked and interact with Nrf2/HO-1 signals. ERK and Akt were phosphorylated at 1–3 h in the presence of 300 μg/mL HHT (Figure 4E). Moreover, the results show that HHT inhibited tBHP-induced apoptosis, but LY294002 (PI3K/Akt inhibitor), PD98059 (MEK1 inhibitor), and compound C (AMPK inhibitor) did not (Figure 4F). This result presents a clue to the mechanism underlying the neuroprotective effects of HHT on the various signaling cascades, including AMPK.

### 2.5. HHT Attenuates Cytotoxicity through Activation of the AMPK Signaling Pathway

**Figure 5:**
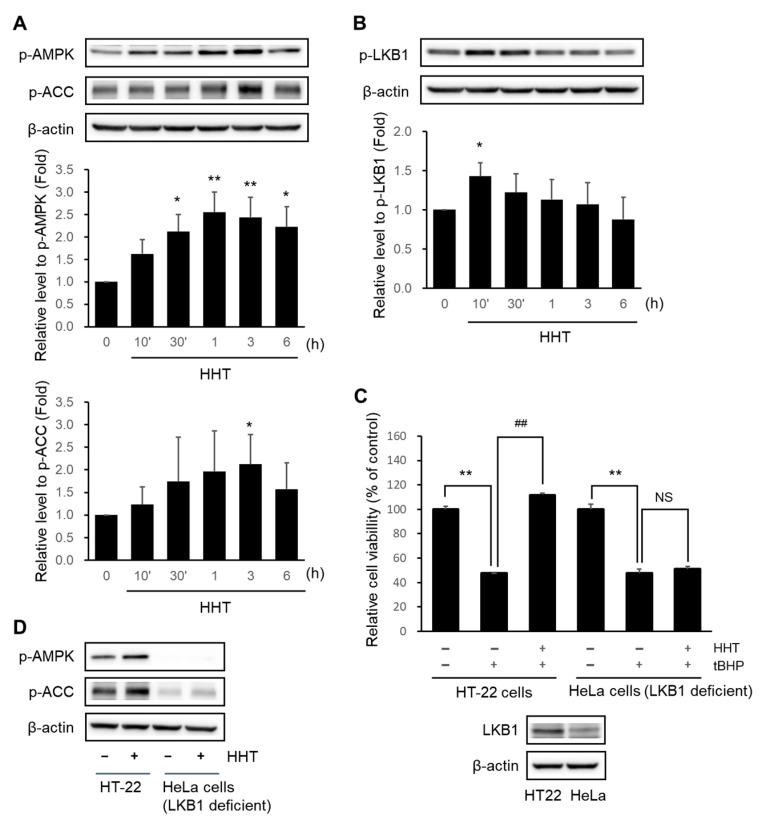
Effects of HHT on the activation of AMPK signaling pathway. Immunoblotting analyses of *p*-AMPK, *p*-ACC (**A**), and *p*-LKB1 (**B**) were performed using HT-22 cell lysates treated with 300 μg/mL HHT for 10 min to 6 h. Expressions of *p*-AMPK, *p*-ACC, and *p*-LKB1 are presented as means ± SDs of three replicate experiments (* *p* < 0.05; ** *p* < 0.01, significant difference compared to the control). (**C**) HT-22 and LKB1-deficient HeLa cells were incubated with 300 μg/mL HHT, followed by MTT assay. Data are presented as means ± SDs of the three replicate experiments (** *p* < 0.01: significant difference compared to control; ^##^ *p* < 0.01: significant difference compared to tBHP). (**D**) Immunoblotting analysis was performed with HT-22 and HeLa cell lysates incubated with 300 μg/mL HHT for 1 h.

AMPK pathway has also been considered as an important pathway similar to Nrf2 because of its potential neuroprotective properties [28]. We verified whether the neuroprotective effects of HHT were affected by the AMPK signaling pathway. At 30 min to 6 h, 300 μg/mL HHT phosphorylated AMPK, and ACC, a downstream kinase of AMPK, was phosphorylated at 3 h (Figure 5A). At 10 min, LKB1, an upstream kinase of AMPK, was also phosphorylated (Figure 5B). Furthermore, HHT attenuated tBHP-induced apoptosis, but did not exert this effect on LKB1-deficient HeLa cells (Figure 5C). The HeLa cells were treated with HHT, but they failed to elicit the expression of phospho-AMPK and phospho-ACC (Figure 5D). These results suggest that HHT attenuates tBHP-induced cytotoxicity by activating the LKB1–AMPK signaling pathway.

### 2.6. Baicalein in HHT Indicates Neuroprotective Effects

**Figure 6:**
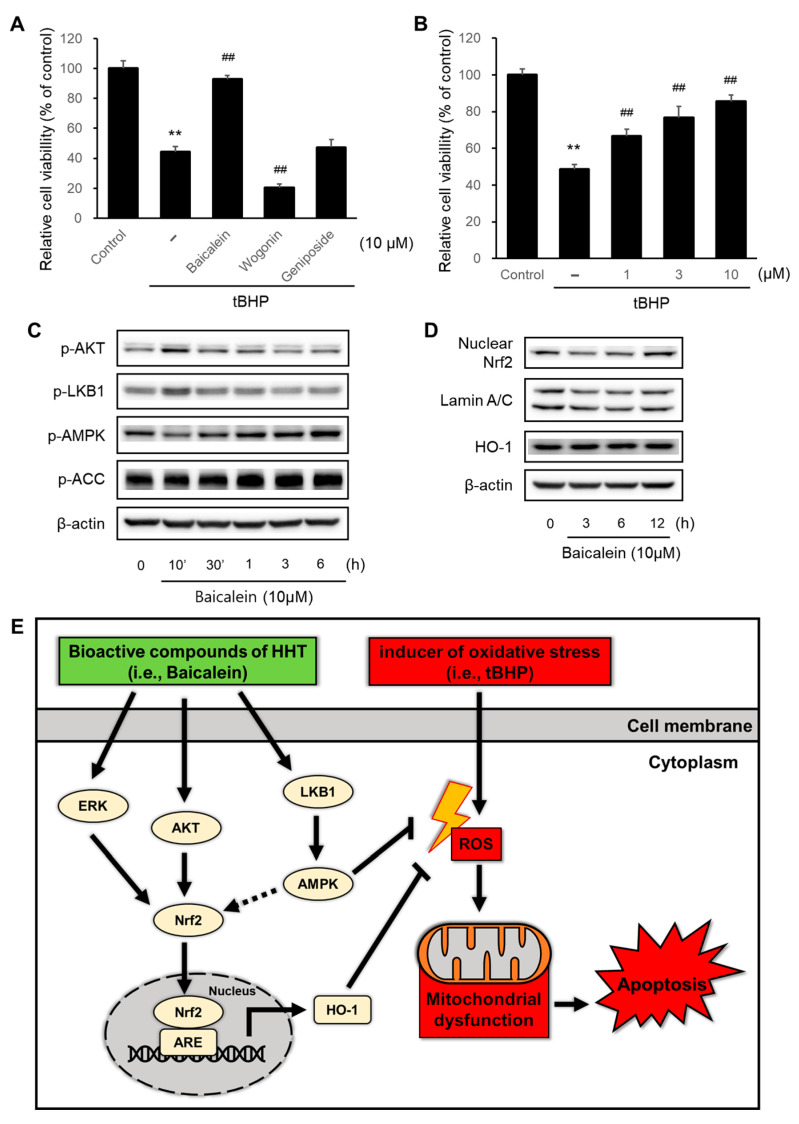
Effects of baicalein, a key compound in HHT, on neuroprotection and activation of AMPK signaling pathway. (**A**) HT-22 cells were treated with 10 μM each of baicalein, wogonin, and geniposide for 1 h, and then incubated with 100 μm tBHP for 6 h, followed by the MTT assay. (**B**) HT-22 cells were treated with baicalein (1, 3, and 10 μM) for 1 h, followed by the next sequence described in Figure 6A. Data are presented as the means ± SDs of three replicate experiments (** *p* < 0.01, significant difference compared to control; ^##^ *p* < 0.01, significant difference compared to tBHP). (**C**) Immunoblotting analysis to confirm activation of the Akt and AMPK signaling pathways was performed with HT-22 cell lysates. Cells were treated with 10 μM baicalein for 10 min to 6 h. (**D**) HT-22 cells were treated with 10 μM baicalein for 3, 6, and 12 h, followed by immunoblotting analysis of Nrf2 in nuclear fractions and HO-1 in whole lysates, respectively. (**E**) Schematic diagram of neuroprotective effects of HHT.

We investigated the neuroprotective effects of the representative compounds (baicalein, wogonin, and geniposide) in HHT. Baicalein (10 μM) significantly suppressed tBHP-induced cytotoxicity, whereas wogonin and geniposide did not (Figure 6A). Baicalein significantly suppressed tBHP-induced apoptosis in a dose-dependent manner (Figure 6B). Baicalein induced the expressions of *p*-Akt, *p*-LKB1, *p*-AMPK, and *p*-ACC (Figure 6C) as well as Nrf2 and HO-1 (Figure 6D). These data suggest that baicalein, a key compound in HHT, exerts a neuroprotective effect by activating the Nrf2/HO-1 signaling pathway and the Akt and AMPK pathways that collaboratively link to this signaling pathway.

## 3. Discussion

Here, we confirmed the effects of HHT on neurological impairments in vivo and in vitro. A 5 familial AD (5xFAD) genetically modified mouse model was used, into which an early onset FAD-causing gene known in humans is inserted, including K670N/M671L (Swedish mutation), I716V (Florida mutation), and V717I (London mutation) APP and PS1 with M146L and L286 mutations. In 5xFAD mice, the accumulation of beta-amyloid in neurons is observed at 1.5 months after birth, and the deposition of amyloid-β plaques in the brain tissue is observed after 2 months [29]. β-amyloid depositions appear in the subiculum area of the hippocampus, and the fifth layer of the cortex in the early stage of the lesion rapidly increases with age and is deposited into most of the hippocampus and cortex by the age of nine months [30]. Behaviorally, it is known that AD lesions appear very quickly in a model, in which memory decline is observed from four months after birth, and neuronal death is observed after nine months. These characteristics make 5xFAD mice a good model for studying the role of intraneuronal Aβ42 in neuronal loss. In this study, we showed that K09 successfully inhibited the pathological process in the 5xFAD mice as assessed by the histological and behavioral indicators. 

During AD development, pathological Aβ and tau accumulate and mislocalize at synapses, leading to synaptic dysfunction and neuronal damage [29,30,31]. Additionally, increased Aβ levels in synapses were found in AD patients and transgenic mice, which is related to the decrease in the synapse density [32]. To identify the therapeutic potential of HHT in AD, six-month old 5xFAD mice with obvious cognitive impairments were administered HHT (50 or 100 mg/kg/day). The administration of HHT for four weeks exerted attenuating effects on the cognitive impairment in 5xFAD mice using the MWM and PA tests (Figure 1). Our results revealed that HHT significantly prevented neuronal impairment and restored neuronal loss in the 5xFAD mouse brains. The administration of HHT reduced the protein levels of Aβ and the number of amyloid plaques. Therefore, HHT may delay amyloid plaque formation more effectively by inhibiting the accumulation of apoptotic cells (Figure 2). In other studies on neurodegenerative diseases, it has been reported that HHT contributed to the amelioration of memory impairment and the reduction of neuroinflammation in APP/PS1 transgenic mice [33], and HHT inhibited the cognitive dysfunction and reduction in the plaque burden in 3XTg-AD mice through a significant decrease in the levels of detergent-soluble and acid-soluble Aβ [34]. Therefore, we could, at least in part, propose that HHT is an effective candidate in the treatment of AD. Furthermore, considering that donepezil possesses the property of anticholinesterase action, the research needs to clarify whether HHT similarly demonstrates inhibition of the breakdown of acetylcholine or not in the future.

Oxidative stress is implicated in various diseases including cardiovascular diseases, chronic kidney disease, chronic obstructive pulmonary disease, sarcopenia, cancer, and neurodegenerative diseases [35]. Oxidative stress refers to high levels of intracellular reactive oxygen species (ROS) caused by an imbalance between ROS formation and the antioxidant defense system [36]. ROS are mediators of cellular signaling pathways involved in various physiological and biological responses, such as antibacterial and antiviral effects, cell growth, proliferation, differentiation, survival, aging, and apoptosis [37]. The overdevelopment of ROS can lead to mutations in cellular genes important for the antioxidant defense system and DNA repair mechanisms [38], which are also responsible for mutations in mitochondrial DNA, the impairment of mitochondrial membrane permeability and mitochondrial respiratory function, and the disruption of Ca2+ homeostasis [39]. Additionally, it was confirmed that conditions of high ROS levels induced the degradation of mitochondrial DNA, the downregulation of antioxidant proteins such as superoxide dismutase 2 (SOD2) and nrf2 by p53, and eventually, apoptosis [40]. This indicates that mitochondrial dysfunction caused by the overproduction of ROS leads to neurodegenerative diseases, including AD, Parkinson’s disease (PD), and Huntington’s disease [41]. Thus, several major pathological processes in AD, including Aβ-induced neurotoxicity, tau-induced neurofibrillary tangles, and mitochondrial dysfunction are associated with oxidative stress involving ROS and reactive nitrogen species [42]. Moreover, tBHP, a membrane-permeant oxidant compound and inducer of oxidative stress, is known to induce apoptosis by disrupting the mitochondrial permeability transition with an excessive production of ROS in neuronal cell lines and rat primary neurons [43,44]. HT22 cells serve as an in vitro model to elucidate the mechanisms of cognitive deficits in Alzheimer’s disease [45]. Therefore, in this study, we adopted an in vitro model of oxidative stress induced by tBHP in HT22 cells. Our results showed the antioxidant effects of HHT in neuronal cells, reducing cell death by inhibiting ROS generation and mitochondrial damage (Figure 3). In other studies, the validated antioxidant sulforaphane, an Nrf2 agonist, also improved the cell viability in HT22 cells by inhibiting ROS generation [46].

Nrf2 is involved in the control of several programmed biological functions such as stem cell regulation, autophagy, inflammasome signaling, unfolded protein response, and mitochondrial biogenesis [47]. Nuclear factor erythroid 2-related factor 2, a major sensor of oxidative stress, counteracts oxidative stress by controlling redox, thereby affecting the expressions of numerous genes that encode proteins involved in cellular redox balance, detoxification, stress, and metabolism [48]. Under basal conditions, Nrf2 is localized in the cytoplasm by cytoplasmic Kelch-like ECH-associated protein 1 (Keap1) and is continuously ubiquitinated by Cullin 3 to undergo proteasomal degradation. Under oxidative stress, Nrf2 is released due to the disruption of the keap1–cullin 3 interaction through a conformational change of the primary redox sensor, Keap1, and translocated into the nucleus without ubiquitination. In the nucleus, Nrf2 heterodimerizes with one of the small Maf proteins, and the heterodimer binds to an antioxidant response element (ARE) to initiate the transcription of target genes such as phase II detoxifying enzymes [49]. Phase II enzymes, which are cytoprotective proteins expressed by ARE-induced transcription, include NAD(P)H quinone oxidoreductase 1 (NQO1), glutamate-cysteine ligase (GCL), glutathione S-transferase (GSTs), and heme oxygenase-1 (HO-1) [50]. The activation of this cascade accomplishes the maintenance of cellular redox homeostasis and robust protection against oxidative stress and related disorders, which has also been demonstrated in in vivo and in vitro neurological disease models [51]. Additionally, the Nrf2 pathway activity affects the inhibition of ROS and the reduction of Aβ deposits and tau phosphorylation in AD animal models and human cell lines expressing AD pathology [52]. Accordingly, it is becoming increasingly clear that Nrf2 is a promising target for the treatment of neurological diseases [53]. Among Nrf2-ARE-induced enzymes, HO-1 is a major anti-inflammatory and antioxidant enzyme [54]. HHT significantly induced the nuclear translocation of Nrf2 and the expression of its target gene, HO-1, in vitro (Figure 6). 

AMPK, an essential cellular energy sensor, is activated in response to various conditions that lower the cellular energy status, such as nutrient starvation (particularly glucose), hypoxia, exercise, and exposure to toxins that disrupt the mitochondrial respiratory chain [55]. Additionally, AMPK is phosphorylated by LKB1, the main upstream kinase that responds to stimuli, which increases AMP and ADP levels (oxidative stress). Activated AMPK promotes the acute regulation of glycolysis, glucose uptake, fatty acid oxidation, and lipid uptake and release, while inhibiting gluconeogenesis, glucose storage, and fatty acid synthesis [56]. Furthermore, AMPK acts as a signaling system to maintain mitochondrial health by regulating mitochondrial fission, mitophagy, and biogenesis, and also regulates factors involved in the transcription of autophagy and lysosomal genes [57]. The interplay (beyond simple parallel activation) of AMPK and Nrf2 signaling by small molecules regulates cellular homeostasis and relieves/prevents various diseases [58]. Additionally, Nrf2 activation dependent on AMPK activation by berberine has been demonstrated to exhibit anti-inflammatory effects [25]. Specifically, in a model of SH-SY5Y cells with neuronal injury caused by mitochondrial fragmentation due to lead-induced ROS accumulation, metformin exhibited a protective effect against lead toxicity by enhancing Nrf2/HO-1 through AMPK activation [26]. The activation of AMPK by cajaninstilbene acid regulates Nrf2, resulting in neuroprotective effects by inhibiting mitochondrial dysfunction in the tBHP-injured SH-SY5Y cell model [27]. In this study, HHT induced Nrf2 as well as AMPK pathways, which had important roles in the antioxidative effects of HHT (Figure 4 and Figure 5). 

In addition, in our study, baicalein exhibited neuroprotective effects by activating the Nrf2/HO-1 signaling pathway through the Akt and AMPK pathways (Figure 6). To date, there are numerous papers showing the antioxidant activity of baicalein. However, studies that investigated the effects of baicalein in a tBHP-induced oxidative stress model in neuronal cell lines are scarce, and the number of studies focusing on the mechanism of the LKB1–AMPK pathway collaboratively linked with the Nrf2/HO-1 pathway are limited. This scarcity of research underscores the novelty of our study and raises anticipation for future research. Baicalein has key pharmacological properties, such as the ability to reduce oxidative stress and inflammation, inhibit disease-specific amyloid protein accumulation and excitotoxicity, activate neurogenesis and differentiation, and antiapoptotic effects [59]. Other studies have shown that baicalein alleviates endothelial oxidative dysfunction by upregulating AMPK and inhibiting PKC and NF-κB signaling pathways [60]. Moreover, baicalein increases the expression of antioxidant enzymes by activating Nrf2, thereby exerting a mitochondrial protective effect by preventing chemically-induced apoptosis [61]. In a traumatic brain injury mouse model, baicalein improved the neurological function and reduced brain edema, oxidative stress, and apoptosis by increasing the expressions of HO-1 and NQO-1 via the activation of Akt/Nrf2 pathways [62]. Therefore, these results reveal that baicalein is a promising therapeutic compound to ameliorate AD through its neuroprotective efficacy against oxidative stress-induced damage. However, it is necessary to determine in subsequent studies whether baicalein, along with the other constituents of HHT, demonstrates synergistic or antagonistic properties. 

## 4. Materials and Methods

### 4.1. Chemicals and Reagents

Anti-Aβ1-42 was purchased from Proteintech (Rosemont, IL, USA). Anti-Nrf2 was purchased from Santa Cruz Biotechnology (Dallas, TX, USA). Anti-lamin A/C, anti-HO-1, anti-phospho-ERK1/2, anti-phospho-Akt, anti-phospho-AMPKα, anti-phospho-LKB1, ACC, anti-β-actin, anti-caspase-3, anti-poly (ADP-ribose) polymerases (PARP), and anti-B-cell lymphoma-extra-large (Bcl-xL) were purchased from Cell Signaling Technology (Danvers, MA, USA). HRP-conjugated anti-mouse IgG and anti-rabbit IgG were obtained from Enzo Life Sciences (Farmingdale, NY, USA). Tert-butyl hydroperoxide, 3-(4,5-dimethylthiazol-2-yl)-2,5-diphenyl-tetrazolium bromide (MTT), propidium iodide (PI), calcein-AM, PD98059, LY294002, 2′,7′-dichlorofluorescein diacetate (DCFH-DA), rhodamine 123 (Rh123), and Harri’s hematoxylin and eosin (H–E) were purchased from Sigma-Aldrich (St. Louis, MO, USA). A 3,3′-diaminobenzidine tetrahydrochloride (DAB) detection kit was sourced from Ventana Medical Systems (Tucson, AZ, USA). A in situ cell death detection kit and fluorescein were purchased from Roche Diagnostics (Rotkreuz, SWI). DAPI was obtained from Vector Laboratories (Burlingame, CA, USA). Compound C was purchased from Calbiochem (San Diego, CA). HHT was used as the product Kracie K-09^®^, manufactured by Kracie Pharma, Ltd. (Minato-ku, Tokyo, Japan). Baicalein (in DMSO, solubility: 54 mg/mL), wogonin (in DMSO, solubility: 56 mg/mL), and geniposide (in DMSO, solubility: 78 mg/mL) were obtained from the Korea FDA.

### 4.2. Treatment of HHT in 5xFAD Mice

All animal experiments were conducted at the Korea Institute of Oriental Medicine-Daegu according to US guidelines (NIH publication #83-23, revised in 1985), and the procedures were also approved by the Korea Institute of Oriental Medicine–Daegu Institutional Animal Care and Use Committee (KIOM-IACUC, reference number 21-095). 5xFAD mice were used to perform animal experiments, while age-matched mice of the same background were used as controls and were obtained from Jackson Laboratory (Bar Harbor, ME, USA). All the animals were housed in a facility at Northwest A&F University under standard conditions (12/12 light–dark cycles, temperature of 22 ± 2 °C, and humidity of 50 ± 15%). The experimental mice were separated into one cage. A standard diet, including pure water and AIN-93 M (TROPHIC Animal Feed High-Tech Co., Ltd., Nantong, China) was provided to all the animals. After the experimental mice were fed for six months, the 5xFAD and wild-type (WT) mice were freely assigned to five groups based on their genotype, and each treatment was administered for 4 weeks (n = 8/group): (i) WT; (ii) 5xFAD control (vehicle); (iii) 5xFAD mice treated with HHT 50 mg/kg; (iv) 5xFAD mice with HHT 100 mg/kg treatment; and (v) 5xFAD mice treated with donepezil 5 mg/kg.

HHT is a product formulated from Kracie Pharma, Ltd., with the manufacturer typically recommending a daily intake of 6.0 g for an average adult (approximately 60 kg); this corresponds to 100 mg/kg. Using this as the base, we provided the mouse dosage equivalent to the human clinical dose.

### 4.3. Morris Water Maze Test

The MWM test was used to investigate the memories and learning abilities of all the mice. The water maze consisted of a 1.2 m diameter rubber pool filled with opaque water (reverse osmosis water diluted with white paint) and a 10 cm diameter hidden platform in the northeast quadrant of the rubber pool. An overhead video camera connected to the SMART video tracking software (Panlab, Barcelona, Spain) was used to track and record them swimming. The mice underwent four habituation training sessions on day zero. The platform was visible (2 cm above the water surface), and the water was undyed. The test trials were conducted for five consecutive days (days 1–5). These behavioral tests were conducted as previously described [31].

### 4.4. Passive Avoidance Test

The PA test was performed using the Shuttle Box Avoidance Basic Test Package (Med Associates Inc., Fairfax, VT, USA). The apparatus was divided into two rooms (one illuminated and one darkened) that were connected via an automatic door. The darkened room was equipped with a grid floor through which an electronic shock could be delivered. The step-through latency times for the mice moving from the illuminated room to the darkened room were recorded for a maximum of 3 min. The PA test was performed as previously described [63].

### 4.5. Tissue Processing

The brain was removed from the cerebellum and brain stalk, and other tissues were collected. The tissues were then placed into a vial containing 4% paraformaldehyde in PBS (pH 7.4) for 24 h at room temperature (n ≥ 3 for H–E and immunochemical staining) until analysis. The prepared tissues were fixed for more than 24 h before dehydration with gradient alcohol. The samples were sequentially soaked in alcohol–benzene and xylene and then embedded in paraffin. The wax-soaked tissues were cut into 5 μm thick slices. Finally, the finished pieces were stored at room temperature.

### 4.6. Hematoxylin and Eosin

Wax pieces of brain tissue were prepared. Initially, different sections were dewaxed using xylene (twice, 20 min each) and gradient ethanol (100% twice, 10 min; 95%, 90%, 80%, 70%, and 50% once, 5 min). After washing with water, wax slices were stained with H–E. Finally, all the pieces were examined using an optical microscope (Olympus).

### 4.7. Immunohistochemical Staining (IHC)

Fixed brain sections were immersed in xylene and gradient ethanol for deparaffinization and rehydration, respectively. Briefly, the brain sections were immersed in xylene and rehydrated in 100%, 95%, 90%, 80%, 70%, and 50% ethanol. The sections were incubated in 3% H_2_O_2_ for 30 min to block endogenous peroxidase, followed by incubation with 5% normal goat serum for 1 h. After blocking, the sections were incubated overnight at 4 °C with primary antibodies (Aβ1-42) and incubated with peroxidase-conjugated secondary antibody for 1 h. The same washing step was repeated using PBS, and the 3,3′-diaminobenzidine tetrahydrochloride was used to dye the prepared samples. Hematoxylin was used to redye, and neutral resin was used to block the brain slices. Finally, images were acquired from stained sections using an optical microscope (Olympus). Representative images of the hippocampus were obtained.

### 4.8. Terminal Deoxynucleotidyl-Transferase-Mediated dUTP Nick End Labeling (TUNEL) Staining

After all the experiments were completed, the fixed brains were processed by embedding them in paraffin blocks, and the brains (5 µm) were sectioned. Apoptotic cells in the cortex were determined using TUNEL. Terminal deoxynucleotidyl transferase dUTP nick end labeling staining was performed six times according to the manufacturer’s protocol (R&D Germany), and TUNEL-positive cells emitted a green, fluorescent color and were quantified using fluorescence microscopy (Olympus BX53; Olympus Corporation) at magnifications of ×100 and ×200, and five fields for each section were examined from the ischemic cortex. The average percentage of TUNEL-positive cells among the total number of cells was determined. The nuclei were stained with DAPI.

### 4.9. Cell Culture

HT-22 was kindly provided by Dr. Sung Hwan Ki (Chosun University, Gwangju, Korea), and HeLa cells were obtained from the American Type Culture Collection (ATCC, Rockville, MD, USA). The cells were cultured in a high glucose, Dulbecco’s modified Eagle’s medium supplemented with 10% fetal bovine serum, 50 μg/mL streptomycin, and 50 units/mL penicillin. The cell culture environment was 37 °C with 5% CO_2_ in a humidified atmosphere. Prior to all experiments, cells were subjected to starvation in serum-free minimum essential medium (MEM) for 12 h.

### 4.10. MTT Assay

HT-22 cells were seeded in each well for 24 h at a density of 1 × 10^4^ in a 48-well plate, followed by incubation in serum-free MEM for 12 h. Cells were incubated with 3, 10, 30, 100, and 300 μg/mL HHT for 1 h, followed by treatment with 100 μM tBHP for 6 h. The staining of cells was performed for 2 h using 0.25 mg/mL MTT, and then the medium was suctioned. Formazan crystals, formed by an MTT tetrazolium reduction by dehydrogenase in the mitochondria, were dissolved in 300 μL of dimethyl sulfoxide. The dissolved formazan crystals were analyzed for absorbance at 540 nm using an enzyme-linked immunosorbent assay (ELISA) microplate reader (Tecan, NC, USA).

### 4.11. Measurement of Reactive Oxygen Species

2′,7′-dichlorofluorescein diacetate (DCFH-DA), a fluorogenic dye, was used to quantify cellular ROS. HT-22 cells were seeded in each well for 24 h at a density of 0.4 × 10^4^ in a 96-well black plate, followed by incubation in serum-free MEM for 12 h. Cells were treated with 300 μg/mL HHT for 1 h and then incubated with 100 μM tBHP for 6 h, followed by staining with 10 μM DCFH-DA for 1 h. 2′,7′-dichlorofluorescein diacetate uptake into cells was deacetylated by cellular esterases to form nonfluorescent compounds. It was then oxidized by ROS to 2′,7′-dichlorofluorescein (DCF), which exhibited a high fluorescence. The fluorescence levels of DCF were analyzed using an ELISA microplate reader.

### 4.12. Measurement of Mitochondrial Membrane Potential

The mitochondrial membrane potential was assessed using flow cytometry after staining with rhodamine 123 (Rh123), a mitochondrial-specific green, fluorescent dye. In 6-well plates, HT-22 cells were seeded in each well for 24 h at a density of 1 × 10^5^, followed by incubation in serum-free MEM for 12 h. Cells were treated with 300 μg/mL HHT for 1 h, and then incubated with 100 μm tBHP for 6 h, followed by staining with 0.05 μg/mL Rh123 for 1 h. Cells were harvested using trypsin, and 10,000 cell populations were recorded to observe the MMP.

### 4.13. Immunoblot Analysis

HT-22 cells were seeded in a 60 Ø dish at a density of 3 × 10^5^, and then HHT was treated at the concentration or time described in each figure. RIPA buffer was used at 4 °C to prepare cell lysates, and a BCA assay kit (Thermo Fisher Scientific Inc., Waltham MA, USA) was used for the protein quantification of lysates. The quantified proteins were sorted by sodium dodecyl sulfate-polyacrylamide gel electrophoresis, and the separated proteins were transferred to an immunoblot PVDF membrane. The protein-bound membrane was incubated with primary and secondary antibodies, and the membrane was reacted with a chemiluminescence reagent and detected using a ChemiDoc image analyzer (Vilber Lourmat, France).

### 4.14. Statistical Analysis

All the results are presented as the means ± SDs or SEMs. A one-way analysis of variance using GraphPad Prism version 5.03 for Windows (GraphPad Software Inc., San Diego, CA, USA) was performed for multi-group comparisons. T-tests were applied to analyze the significance of the variations among the groups. Statistical significance was regarded as *p* < 0.05, *p* < 0.01, and *p* < 0.001.

## 5. Conclusions

In a 5xFAD mouse model, our research confirmed that HHT alleviates cognitive impairments, reduces the protein levels of Aβ, and prevents neuron impairment. In HT-22 cells, HHT attenuated tBHP-induced cytotoxicity, ROS generation, and mitochondrial dysfunction. It was verified that HHT exerts a neuroprotective effect by activating signaling pathways such as LKB1/AMPK, Nrf2, MAPK/ERK, and PI3K/Akt. In addition, baicalein, a bioavailable compound of HHT, was verified to be a key component. Our study suggests that HHT and its major bioavailable compounds have potential in the prevention and treatment of AD.

## Data Availability

The data presented in this study are available upon request to the corresponding author with appropriate reason.
